# Supplementary material for: Patterns of Intron Gain and Loss in Fungi
Source: PLoS Biol. 2004 Nov 30;2(12):e422. doi: 10.1371/journal.pbio.0020422 (PMC532390; doi:10.1371/journal.pbio.0020422)
Supplement: Table S1 — Also available at http://genes.mit.edu/NielsenEtAl/. (4.3 MB ZIP). [file pbio.0020422.st001.zip › NielsenEtAl/html/893.html]

AN1111.1.NCU00784.1.MG08730.1.FG09695.1


```
 CLUSTAL W (1.82) Multiple Sequence Alignments - Introns Inserted


Sequence 1: NCU00784.1	683 aa
Sequence 2: FG09695.1	737 aa
Sequence 3: MG08730.1	860 aa
Sequence 4: AN1111.1	1187 aa
Alignment Length: 1328 aa
Number Identitical Residues: 126 aa
Alignment Score (without introns) 8129


MG08730.1 	------------------------------------------------------------
NCU00784.1	------------------------------------------------------------
FG09695.1 	------------------------------------------------------------
AN1111.1  	MPKTHQESSRVVAIMKKYLLSLLAAVTYTTAQSVSGSAEGFASGVTGGGGATPVYPADTA
          	 ..: ..::   :  ..   :  :: : :::.: :.::.. ::. :....::.  .:.::

MG08730.1 	------------------------------------------------------------
NCU00784.1	------------------------------------------------------------
FG09695.1 	------------------------------------------------------------
AN1111.1  	ELESLLSSEGEQVIVLTKTYDFTGTSATGTACYSWGTGEGCQLILQDDCGDTPSTTVTYD
          	. .:  ::....    :.: . :.::::.::. : .:.....   ......:.::: : .

MG08730.1 	------------------------------------------------------------
NCU00784.1	------------------------------------------------------------
FG09695.1 	------------------------------------------------------------
AN1111.1  	AAGKTPIPVASDKTLLGVGSEGVIKGKGLSFTDNVSNIIVQNIKITELNPEYVWGGDALT
          	::..:. . ::..:  . .:..  .... : :.. :.   .. . :. ...   ...: :

MG08730.1 	-------------~----------------------------------------------
NCU00784.1	-------------~----------------------------------------------
FG09695.1 	-------------~----------------------------------------------
AN1111.1  	FDGSSNICIDHVE0TSLIGRVHYVFGYNPNSGITLSNNFINGETTYSTSCDGYQYWGLEL
          	 ..::. . .  . ::  .      . ...:. : :..  ...:: :::... .  . . 

MG08730.1 	-----------~------------------------------------------------
NCU00784.1	-----------~------------------------------------------------
FG09695.1 	-----------~------------------------------------------------
AN1111.1  	VGKNDSITFKG1NYLYKTSGRSPALSGSTKFHACNNVWSDNNGHAIEGNEKGQGLFEGNV
          	 ....: : .. .   .::. :.: :.::.  :...  :.... : ........  ... 

MG08730.1 	------------------------------------------------------------
NCU00784.1	------------------------------------------------------------
FG09695.1 	------------------------------------------------------------
AN1111.1  	FQEVATVVSSTFWEAGDLFLSSADGTGNDACASYIGRNCASSIYMNDGGDYTSYTDVSWL
          	 .. ::  :::  .:..   :::..:...:.::  . ..:::   ..... :: :. :  

MG08730.1 	-----~------------------------------------------------------
NCU00784.1	-----~------------------------------------------------------
FG09695.1 	-----~------------------------------------------------------
AN1111.1  	GDWSA1HKGGEKMTSGRITGVWQMYYGLFAFSEDPKLFSIINRLRPSTGGYYENRLVRRA
          	.. ::  ..... ::.  :.  .   .  : :....  :  .   .::..  ..     :

MG08730.1 	--~---MAAKTVCVIG1YGPSGACAAKYLNAELVQGAGHIALNQGYHRFKVTIFETQPRL
NCU00784.1	--~--MAASKKVCIVG1AGPSGLVAAKSLLHDQPKGT-----------FDVTIFEAQNRV
FG09695.1 	--~----MAKTVCIVG1AGPSGLVAAKTLLHNTAPGE-----------FKVTIFDAQKDI
AN1111.1  	VM0PHMAKPKRVAIIG1AGPSGLVTAKTLLRNFPPGT-----------FSAVIFEKKPRI
          	   .    .* *.::*  ****  :** *  :   *            *...**: :  :

MG08730.1 	GGLWPSSPTDA------------------------AGQVHPSMPCNQSRHTVQFSDLAWE
NCU00784.1	GGLWPSRKDDN------------------------AGLVHPLMVANQSKHTVQFSDLAWP
FG09695.1 	GGLWPTSKTDD------------------------GRQVHPFMWSNQSRHTMQFSELAWE
AN1111.1  	GGLWAVDYPSTPYLLRPTPHFSHDYELHCRKQYQGRALVNPWMRTNLSRFSVAFSDLAWE
          	****.    . .    .:.  : . .  . .. ..   *:* *  * *:.:: **:*** 

MG08730.1 	----DNAPEFPRAHDVGRYLARYAATYLPEPAEAEGNTPEALYKRAQGTVEVHRNARVVG
NCU00784.1	----DDAPEFPRAWHIGRYLEAYHDRYCR-------------------DAKIHLGQRVVH
FG09695.1 	----DSDPQLPQGWMVGKYLRRYLERFLTN----------------NENFELKLDTRVES
AN1111.1  	TALGDAVPLFPYAWEVRKYLEKYAETYIP-------------------KECFKLGKRVLS
          	:: .*  * :* .  : :**  *   :                        .: . **  

MG08730.1 	VEPVAVPAGGKPGEAGWDVRV~EGEGS---ARRFDAVVVA~SGFFGRPEIGRVDVGGAGV
NCU00784.1	SELLQPSDQGVEG-AKWKVRT~CSQDGQQLDHLFDYLLVA~SGFFGKPVIPPPVAASGDI
FG09695.1 	AERPQGAVHG------WTVNV~KSDVG-TETQSFDYLLVA~SGFFGKPIVPESLENEKNI
AN1111.1  	VTRRGTEIRAKD----WAVWE0MWRGP------------S1SGHFGSPSIPLIP-GLEDI
          	         .      * *                    : **.** * :        .:

MG08730.1 	PVVPAGQYRDVASLLDAKGKTSGGKGKTSGGKVLVVGSQMSAYEVAGTIARDLSSAVHSP
NCU00784.1	PVVHSSQYRTLTALLEQT--------KGVGRKIVVVGGQMSGVEIAGTVATHLSSALHAP
FG09695.1 	PVIHSSHYRDVKSLLKGR--------SGGG-KILVVGGQMSGVEIAGTIAAHLSSETNSP
AN1111.1  	SSVHSSELQSPDDIGRLLEK------SPKSGKLVVVGGSMSGVETATSLALHLSSLSFRP
          	. : :.. :    :     .      .  . *::***..**. * * ::* .***    *

MG08730.1 	GGVGGIENPERYRVYHVAKRPSWLFPLIGTAT0PSHPRP----RFLPVDLPAYNLSRRQL
NCU00784.1	G-ASPLQHLDQYSIHHISQR------------~---------------------------
FG09695.1 	D-TSSITDADKYSIHHVIQRPIWVFPLYTSPK0PTNKAA----PFLPLDFSSYNLNNRPK
AN1111.1  	G---SSAQQHNYEVWHVGPGPYWVLPTYLPHN~YAKDTEGDAMPFVPLDLSLYDIARRPL
          	.      . ..* : *:   .    .   . .  :    ..:    . . .  .      

MG08730.1 	PLTNNQGILSQQRMREVNYTFSVVLGGDQGDVSPD~LKITDKLA---EEAPYLAMSDSYL
NCU00784.1	---------------------------------PV2LAITNTDT---DQPPYLAVSDTYA
FG09695.1 	PLANTQGHIPEETAKVVHGIYKGVIGTDQSEFSPL~LKTDGTND---DKQPYLAVSEWYT
AN1111.1  	GMTGIAFGPTSQDQNRKRNQFFRDMLGEEYSKVGS~FHLGDNQGREIQRSPWVGIADHYA
          	  :.     ...               .. .     :   ..   . :. *::.::: * 

MG08730.1 	EFVRSGDIVHVPGTVTRVSDRS---ASVENAG-EEINIDDIAAVVSATGWSVKDSLQFLP
NCU00784.1	DFVRSGLISVSRGKLDSLTGDT---VTVTTARGEVSKIDNVAAVVLATGFEAASSISFLP
FG09695.1 	DFVRSGLITLSNGKVETLEGST---AILSDG----TKVADVAAVVAATGFDASPCISYLP
AN1111.1  	EFVRSGAIKTLAGRASSFTNESGLETLNIDTSAGMVRLTEIAAVVMATGFKPSGSLSFLP
          	:***** *    *    . . :. ..      .   .: ::**** ***:.   .:.:**

MG08730.1 	QSIKDVLEFDEADNTHPLVLKFHNTHHPDVANLGFVGFYRSPYWGVIEMQSRFLAKYLGQ
NCU00784.1	SPVQKILSVVPSDLNTTVALAFHGTHHPAIPSLGFVGFYRSPYWGVMEMQARFVTALWAA
FG09695.1 	SDVLQALQFSPKHIDQPVALAFHGTHHPQVPDLGFVGFYRSPYWGVMQMQARFVAALWSD
AN1111.1  	DDVLSTLEYSKDDHFLPLVLDSMSSAHSEIPDLGFVGFYRGAFWGPAELQAQSLAQTWAA
          	. : . *.    .   .:.*   .: *. :..********..:**  ::*:: ::   . 

MG08730.1 	DFRPPAAANPNITTSSESVLEAGQAQVTVPPLANSLKSALEADDCMKKERALRVHRKEHP
NCU00784.1	GGPASSSLPP------------------------RMATALAEDDSIEQTLALRTD-----
FG09695.1 	N-VSPGRNSE------------------------VFKCKLRDDVSIQRTLDLRDD-----
AN1111.1  	ADLENGVSAS-----------------------LSEEEKRSRDGERQQVRDFRDVR--PT
          	     .                                    *   ::   :*      .

MG08730.1 	EAAAQYPMGDYAWLMQEFARALDINMTPPPTATELIPPDGDEPMNILTPSRYPSKGASGQ
NCU00784.1	PRVSQFPMGDYPWLMQEFSRALEIDRIPPLG-EMPRVPPADQSMNILTPARYPAKRAGEA
FG09695.1 	PRVSQFPMGDYPYLMNEIAEALEIQISEPLTPPVLELPHNNLPLDMLTPARYSNPSDDQE
AN1111.1  	SLRGQFPLGDYVGLMESFARRLHRSRFPLGISPDANIRTTGPVVPARYALIDHGTLFEYP
          	   .*:*:***  **:.::. *. .      .        .  :    .           

MG08730.1 	QLDEMSKNMAATRRTVSAALK-DGRFVARAIFRGLQGEWAMDREITSFNDHYGFGSGHYV
NCU00784.1	QRTEATKAIQQTESTAWSGAS-SARFVAKAVFRSLLGEWKLERDLVSRLPTH--PSGNFS
FG09695.1 	SKDAATTSLEQTRKDATDGLT-TSRFVAHAVFRSLLGTWKLERDLISKLPSH--PSGHFS
AN1111.1  	TLKEVEITMKALKSTLLPDPGQASVTTTAAIFRALHGNWGFERIVSHAGGED----IKSC
          	        :   .        . .  .: *:**.* * * ::* :            :  

MG08730.1 	GTAYFYPRKATKIG---LDKAIAEDPAAMALMHEYLYKEKGEFTPVGSNQSFTTWRYYVY
NCU00784.1	GTARFLLRERTRDGREAEHDAALE--KDDDIGLEYLYVEEGMFTASNG-FSFRATRRYVW
FG09695.1 	GTAQFLLREKTTDGLRCAGNPSEDSTNGDELVMEYLYIEEGEFKTSQG-FGFKATRRYVW
AN1111.1  	GKTTFYPRYP----------------SSPMYEAEYLCKEDANDGAAMKTAISVYRHLNTF
          	*.: *  *                         ***  *..   .   .      :  .:

MG08730.1 	RYNEATDEITVWFVDTKDQKSVDYFFHKVELEQISTQGGEENGSDDEDAMARSTNMSDMD
NCU00784.1	RYDEKKDKLSVWFVKTDDQKKADYLFHQVEFVIPNTE-----------------------
FG09695.1 	RYNELKDVLSVWFTKPDDLKRADYLFHDVEFTKP--------------------------
AN1111.1  	RFPGQAG-ISVWFVDGKSCPNSATEFALKLQVEPMGK-----------------------
          	*:    . ::***.. ..       *          .                       

MG08730.1 	SAAVAIHGMSLPNDDMPHWNAKGNHWCPPDQYDLAYRFEFRAVRIPRFNITTYVKGEKKD
NCU00784.1	-----------TEDAPQAWRANAGHLCIDDFYDVDYRFNFKAVNLKDWSLAYRVKGPKKD
FG09695.1 	--------------TEKGWAAKAGHLCIDDYYDVKYNFAFQAVNLKEWQIEYTVKGPKKD
AN1111.1  	------------VTDSGEILVRAVAICESAAKEHTYEFYFDRVAISNWCHRVVWSAKSRD
          	                    ...   *     :  *.* *  * :  :      .. .:*

MG08730.1 	YRSITWFARKTRSEEQE
NCU00784.1	YVIEGTYTR--------
FG09695.1 	YTITGNYTR--------
AN1111.1  	RRQTTWYSRPNDD----
          	      ::* . .
```
